# 2-Aminothiophene Derivative SB-83 Inhibits Trypanothione Reductase and Modulates Cytokine Production in *Trypanosoma cruzi*-Infected Cells

**DOI:** 10.3390/pathogens15010064

**Published:** 2026-01-08

**Authors:** Airton Lucas Sousa dos Santos, Vanessa Maria Rodrigues de Souza, Julyanne Maria Saraiva de Sousa, Raiza Raianne Luz Rodrigues, Mércya Lopes Braga, Maria Gabrielly Gonçalves Da Silva Sousa, Douglas Soares de Oliveira, Mirely Vitória Farias da Silva, Edeildo Ferreira da Silva-Junior, Thaís Amanda de Lima Nunes, Marcos Vinícius da Silva, Ingrid Gracielle Martins da Silva, Karine Brenda Barros-Cordeiro, Sônia Nair Báo, Francisco Jaime Bezerra Mendonça Junior, Klinger Antonio da Franca Rodrigues

**Affiliations:** 1 Infectious Disease Laboratory, Campus Ministro Reis Velloso, Federal University of Delta do Parnaíba, Parnaíba 64202-020, PI, Brazil; sousaairtonlucas@gmail.com (A.L.S.d.S.); rodriguesvanessa745@gmail.com (V.M.R.d.S.); jully.yanne@gmail.com (J.M.S.d.S.); raizzaraianneluz@gmail.com (R.R.L.R.); mercyalbraga@ufdpar.edu.br (M.L.B.); mariagabrielly@ufdpar.edu.br (M.G.G.D.S.S.); douglassoares@ufdpar.edu.br (D.S.d.O.); 2 Research Group on Biological and Molecular Chemistry, Institute of Chemistry and Biotechnology, AC Simões Campus, Federal University of Alagoas, Maceió 57072-970, AL, Brazil; mirely.silva@iqb.ufal.br (M.V.F.d.S.); edeildo.junior@iqb.ufal.br (E.F.d.S.-J.); 3 Laboratory of Immunology and Parasitology, Institute of Biological and Natural Sciences, Federal University of Triângulo Mineiro, Uberaba 38025-180, MG, Brazil; thaisaln13@gmail.com (T.A.d.L.N.); marcos.silva@uftm.edu.br (M.V.d.S.); 4 Microscopy and Microanalysis Laboratory, Department of Cell Biology, Institute of Biological Sciences, University of Brasília, Brasília 70910-900, DF, Brazil; gracilias@gmail.com (I.G.M.d.S.); karine.brenda22@gmail.com (K.B.B.-C.); snbao@unb.br (S.N.B.); 5 Laboratory of Synthesis and Drug Delivery, Department of Biological Sciences, State University of Paraíba, João Pessoa 58071-160, PB, Brazil; franciscojaime@servidor.uepb.edu.br; 6 Center for Basic and Applied Immunology, Dom Delgado University City, Federal University of Maranhão, São Luiz 65065-545, MA, Brazil

**Keywords:** 2-aminothiophenes, trypanothione reductase inhibitor, reactive oxygen species, electron microscopy, *Trypanosoma cruzi*, chagas disease

## Abstract

Chagas disease remains a significant neglected tropical disease that predominantly affects vulnerable populations in rural, low-income areas of Latin America. The management of this condition is severely hindered by the limitations of current therapies, which are characterized by substantial toxicity, diminished efficacy during the chronic phase, and the emergence of parasitic resistance. Given the promising activity of SB-83 (a 2-aminothiophenic derivative) against *Leishmania* spp., the present study sought to evaluate its trypanocidal activity against *Trypanosoma cruzi*. The results showed that SB-83 exhibited potent inhibitory effects on the epimastigote forms of *T. cruzi* (IC_50_ = 6.23 ± 0.84 μM), trypomastigotes (EC_50_ = 7.31 ± 0.52 μM) and intracellular amastigotes (EC_50_ = 5.12 ± 0.49 μM). Furthermore, the cellular proliferation assay results indicated CC_50_ values of 77.80 ± 2.05 µM for LLC-MK2 CCL-7 and 24.21 ± 1.2 µM for Vero CCL-87, with a selectivity index above 10 for LLC-MK2 cells. In addition, the compound increased TNF-α, IL-12, nitric oxide, and ROS while decreasing IL-10. Moreover, in silico and in vitro assays confirmed its binding to trypanothione reductase, disrupting redox balance. Flow cytometry further revealed apoptosis induction in trypomastigotes, whereas electron microscopy showed cellular disruption and organelle disorganization. Therefore, SB-83 demonstrated potent activity against the *Tc*I-resistant strain linked to Chagas cardiomyopathy at non-toxic concentrations for host cells, supporting its potential as a therapeutic candidate.

## 1. Introduction

Chagas disease, also known as American trypanosomiasis, is caused by the protozoan *Trypanosoma cruzi*, an obligate intracellular parasite first described by Carlos Chagas in 1909, belonging to the order Kinetoplastida and family Trypanosomatidae [1]. It is recognized as one of the Neglected Tropical Diseases (NTDs) by the World Health Organization (WHO) and is of great global relevance, affecting approximately 6 to 7 million people, especially in low-income tropical and subtropical areas. Transmission mainly occurs through contact with feces of infected triatomines, but can also occur through blood transfusions, organ transplants, contaminated food and congenital transmission [2].

Chagas disease was historically prevalent in rural areas where poor housing conditions favored the proliferation of triatomine vectors. However, urbanization and migration processes have contributed to the expansion of cases in urban settings and, more recently, to the spread of the disease to non-endemic countries, including the United States and several countries in Europe and Asia [3]. In Brazil, an estimated 1.1 to 4.6 million people are infected with *Trypanosoma cruzi*, with approximately 25.4 million individuals living in areas at risk of transmission, highlighting Chagas disease as a major public health problem [4].

Chagas disease has two main clinical phases: the acute phase, which may be asymptomatic or present non-specific symptoms, and the chronic phase, marked by serious complications such as Chagas cardiomyopathy and digestive megasyndromes. The severity of the clinical picture is directly related to the host’s immune response and the parasite load [5]. Despite this, current treatment is limited to two drugs, nifurtimox and benznidazole, which in addition to presenting efficacy restricted to the acute phase, are associated with severe adverse effects, low adherence and important contraindications, such as use in pregnant women [6].

Given these limitations, there is an urgent need to develop more efficient, safe, and accessible therapies which are effective in all phases of the disease. In this context, thiophene-derived compounds have gained prominence due to the presence of a thiophene ring in their structure, which significantly contributes to the chemical stability and bioactivity of the molecule, enabling its interaction with different cellular targets [7]. Among these compounds, SB-83 stands out as a 2-aminothiophene derivative that has demonstrated activity at low concentrations against resistant *Leishmania (Leishmania) amazonensis* strains in vitro, inducing cell death by apoptosis and modulating the immune response [8].

Rodrigues et al. [9] recently observed similar effects against *Leishmania* (*Leishmania*) *infantum*, the etiological agent of visceral leishmaniasis, reinforcing the potential of SB-83 as an anti-trypanosomatidae agent. Furthermore, it has demonstrated anticancer properties, promoting cell cycle arrest and increased expression of pro-apoptotic proteins and cell proliferation inhibition [10,11,12]. Considering that *Leishmania* spp. and *T. cruzi* belong to the same family (Trypanosomatidae), this study proposes to investigate the in vitro activity of SB-83 against *T. cruzi*, investigating its direct and indirect effects, as well as its toxicity in epithelial cells.

## 2. Materials and Methods

### 2.1. Reagents

Liver Infusion Tryptose (LIT; NaCl, 4 mg/mL; Na_2_HPO_4_·12H_2_O, 11.6 mg/mL; KCl, 0.4 mg/mL; glucose, 2.2 mg/mL; tryptose, 5 mg/mL; liver infusion, 5 mg/mL; hemin, 25 mg/mL), Dulbecco’s Modified Eagle’s Medium (DMEM), 3-(4,5-dimethylthiazol-2yl)-2,5-diphenyl tetrazolium bromide (MTT), stabilized antibiotic solution (penicillin 10,000 U/mL; streptomycin 10 μg/mL), stabilized antibiotic-antimycotic solution (penicillin 10,000 U/mL; streptomycin 10 μg/mL; amphotericin B, 25 μg/mL), trypan blue, sodium cacodylate, Permount resin, and lead citrate were purchased from Sigma-Aldrich (St. Louis, MO, USA). Dimethyl sulfoxide (DMSO, 99%) and sodium dodecyl sulfate (SDS) were obtained from Mallinckrodt Chemicals (St. Louis, MO, USA). Fetal bovine serum (FBS) was provided by Cultilab (São Paulo, SP, Brazil). Benznidazole was purchased from Laboratorio Farmacêutico do Estado de Pernambuco (Recife, PE, Brazil). Glutaraldehyde, osmium tetroxide, uranyl acetate, and Spurr resin were obtained from Electron Microscopy Sciences (Hatfield, PA, USA). The FITC-Annexin V apoptosis detection kit with propidium iodide (PI) was purchased from Biolegend (San Diego, CA, USA).

### 2.2. Compound Obtainment

**Figure 1:**
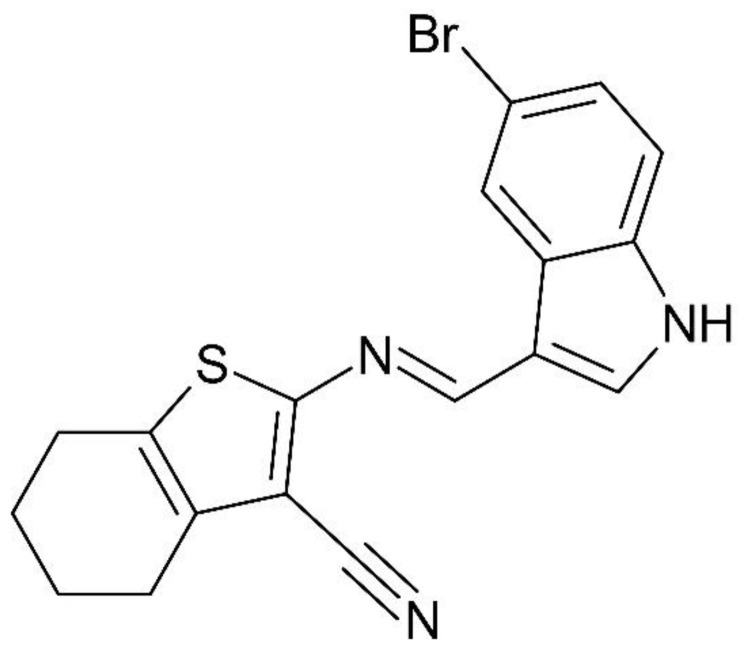
Chemical structure of SB-83.

The compound, 2-{[(5-Bromo-1*H*-indol-3-yl)-methylene]amino}4,5,6,7-tetrahydrobenzo[*b*]thiophene-3-carbonitrile, called SB-83 (Figure 1), was resynthesized according to the methodology described by Souza et al. [13]. In summary, the intermediate 2-amino-4,5,6,7-tetrahydrobenzo[*b*]thiophene-3-carbonitrile was obtained by the Gewald reaction [14]. Then, this intermediate was condensed with 5-bromo-indole-3-carboxaldehyde in ethanolic medium under reflux, resulting in the formation of the SB-83 compound. As SB-83 was previously described, its chemical structure was confirmed after purification by comparison with reported physicochemical data (R.f. and melting point). Its purity was determined by HPLC LC-20A Prominence, Shimadzu (Kyoto, JPN) and it was over 99.9% (chromatogram available in the Supplementary Material). Stock solutions of 20 mg/mL were prepared in DMSO and diluted in specific culture media, ensuring a final concentration of DMSO ≤ 0.5%.

### 2.3. Maintenance of Parasites and Cell Cultures

Epimastigote forms of *T. cruzi*, Colombian strain (DTU TcI), were cultured in LIT medium, pH 6.9, supplemented with 10% fetal bovine serum (FBS) and 1% antibiotic solution (penicillin 10,000 IU/mL and streptomycin 10 mg/mL). The parasites were maintained at 26 °C in a biological oxygen demand (BOD) incubator SolidSteel (São Paulo SP, Brazil) [15]. LLC-MK2 (ATCC CCL-7) cell lines derived from rhesus monkey kidney, Vero (ATCC CCL-81) cell lines derived from African green monkey kidney, and RAW 264.7 macrophages (ATCC TIB-7) were cultured in DMEM, supplemented with 10% FBS and 1% antibiotic solution. The cells were maintained at 37 °C under a humidified atmosphere with 5% CO_2_ in culture bottles [16].

The trypomastigote forms were obtained from the supernatant of previously infected Vero CCL-81 cell cultures and maintained in a cell culture bottle with DMEM supplemented with pH 7.2. The cultures were maintained in an incubator at 37 °C with 5% CO_2_ until cell confluence was reached [17]. After reaching confluence, the supernatant containing the trypomastigote forms was carefully collected and subjected to centrifugation (3500 rpm × 10 min) to obtain a parasite concentrate. The resulting pellet, rich in trypomastigotes, was used directly in the biological assays. When necessary, this concentrate was stored in liquid nitrogen in a cryoprotectant solution containing 90% FBS and 10% DMSO. After removing the supernatant, adherent cells were washed twice with PBS and cultured in fresh medium. The persistence of infected cells and residual trypomastigotes in the supernatant allowed the infection cycle to continue, and new cell aliquots (1 × 10^4^ cells per flask) were added as needed to prevent cell density reduction.

### 2.4. Anti-Trypanosoma cruzi Activity Against Epimastigote and Trypomastigote Forms

The anti-*T. cruzi* activity of SB-83 was evaluated against epimastigote and trypomastigote forms in 96-well plates. For epimastigotes, parasites in the exponential growth phase were adjusted to a density of 1 × 10^7^ parasites/mL, distributed into 96-well plates, and incubated with SB-83 diluted in LIT medium to obtain final concentrations ranging from 3.12 to 100 µM at 26 °C in a B.O.D. incubator for 72 h. For trypomastigotes, parasites were adjusted to a density of 1 × 10^7^ parasites/mL, plated in 96-well plates, and incubated with SB-83 diluted in DMEM (3.12–100 µM) at 37 °C in a 5% CO_2_ atmosphere for 72 h.

After incubation, 10 µL of MTT solution (5 mg/mL) was added to the wells, followed by a new incubation for 4 h. Then, 50 µL of SDS were added to solubilize the crystals, with overnight incubation. Absorbance was measured in an ELISA microplate reader BioSystems model Elx800 (Curitiba, PR, Brazil) at 540 nm. Benznidazole at concentrations of 3.12 to 200 µM was used as a positive control [18].

### 2.5. Evaluation of SB-83 Cytotoxicity Against Epithelial Cells

The cytotoxic profile of SB-83 was evaluated using the MTT assay. Cells of the LLC-MK2 (CCL-7) and Vero (CCL-81) cell lines were seeded in 96-well plates with 100 µL of complete DMEM containing a cell density of 1 × 10^5^ cells/well. The plates were incubated for 4 h at 37 °C in an atmosphere with 5% CO_2_ to allow cell adhesion. The medium was then removed, and the wells were washed twice with PBS to eliminate non-adhered cells.

Next, 100 µL of complete DMEM containing increasing concentrations of SB-83 (1.56 to 200 µM) were added to the wells, and the plates were incubated for 72 h under the same conditions. Benznidazole (3.12–200 µM) was used as a positive control. After treatment, 10 µL of MTT was added to each well, followed by incubation for another 4 h. The medium was subsequently removed, and 100 µL of DMSO was added to solubilize the formazan crystals. Absorbance was measured in an ELISA microplate reader at 540 nm [19]. The selectivity index for each treatment was calculated by dividing the CC_50_ with the IC_50_.

### 2.6. Anti-Trypanosoma cruzi Activity Against Intramacrophagic Amastigotes

First, RAW 264.7 macrophages (1 × 10^5^ cells/well) were cultured in 24-well plates containing glass coverslips (13 mm) and incubated at 37 °C with 5% CO_2_ for cell adhesion. After 4 h the wells were washed three times with PBS. Next, the cells were infected with 1 × 10^6^ trypomastigotes/well (ratio 10:1) and incubated for 24 h. The wells were washed again to remove non-internalized parasites and treated with SB-83 (3.12–25 μM) or benznidazole (200 μM, positive control) for 72 h. After incubation, the coverslips were fixed, stained with a panoptic kit and mounted with Permount resin. Microscopic analysis was performed on 300 macrophages/coverslip. The infection index was calculated by multiplying the number of infected macrophages by the mean number of amastigotes per cell [20]. The supernatant was stored in liquid nitrogen for cytokine and nitric oxide analyses.

For the epimastigote recovery assay, 96-well plates were incubated for 72 h in SB-83-treated (3.12–25 μM). The medium was replaced with complete DMEM without the compound to allow amastigotes to differentiate into trypomastigotes. After 72 h, 100 μL of the supernatant containing trypomastigotes was transferred to plates with LIT medium and incubated at 26 °C for 120 h in a BOD chamber. Epimastigote quantification was performed by counting in a Neubauer chamber [21].

### 2.7. Evaluation of the Immunomodulatory Profile of SB-83 in Macrophages Infected with Trypanosoma cruzi

#### 2.7.1. Cytokine Dosage

Cytokine quantification in supernatants of infection assays was performed by sandwich ELISA, following the manufacturer’s specifications (eBioscience, San Diego, CA, USA). ELISA plates (NUNC-ImmunoTM, Sigma-Aldrich, St. Louis, MO, USA) were sensitized with capture antibodies and incubated at 4 °C for 18 h. After washing with PBS-Tween 0.05%, the wells were blocked with PBS-10% FBS and incubated for 1 h at room temperature. Culture supernatants and recombinant cytokine standard curves were added to the plates, followed by incubation at 4 °C for 18 h.

After another wash, biotinylated detection antibody was added and incubated for 1 h at room temperature. Then, avidin conjugated with peroxidase (avidin-HRP) was applied and incubated for 30 min. The reaction was revealed with tetramethylbenzidine (TMB) and hydrogen peroxide and was stopped with 1N sulfuric acid after 15 min. Absorbance was measured at 450 nm, and the TNF-α, IL-6, IL-10, and IL-12 concentrations were determined by interpolation with the standard curve. LPS and IFN-γ were used as positive controls.

#### 2.7.2. Nitric Oxide (NO) Dosage

NO production was assessed by the Griess method [22]. Aliquots of 100 μL of the supernatants were transferred to 96-well plates, followed by adding 100 μL of Griess reagent. Absorbance was measured at 540 nm after incubation for 10 min at room temperature. Nitrite concentration was calculated by interpolation with a standard curve of sodium nitrite (NaNO_2_). LPS and IFN-γ were used as positive controls.

#### 2.7.3. Measurement of Reactive Oxygen Species (ROS)

ROS levels were quantified by fluorimetric assay with H_2_DCFDA dye [23]. Macrophages were cultured in 96-well plates (1 × 10^6^ cells/well) in supplemented DMEM and incubated at 37 °C with 5% CO_2_ for 4 h. After adhesion, the cells were infected with *T. cruzi* trypomastigotes (5:1 ratio, 1 × 10^5^ parasites/well) for 24 h. Treatment with SB-83 (3.12–25 μM) was maintained for 72 h, with LPS and IFN-γ as positive controls. Then, H_2_DCFDA (20 μM) was added, and the plates were incubated at 37 °C for 30 min in the dark. Fluorescence was quantified in a FLx800 spectrofluorometer (excitation: 485 nm; emission: 528 nm).

### 2.8. Evaluation of the Effect of SB-83 on Trypanothione Reductase (TR) Activity

The inhibitory activity of the SB-83 compound was evaluated against a concentrated fraction of the TR enzyme. The fraction containing TR was obtained from *T. cruzi* in the trypomastigote form (1 × 10^7^ parasites), centrifuged at 1500 rpm for 10 min. The pellet was resuspended in a solution of HEPES (40 mM) and EDTA (1 mM) and subjected to lysis using a homogenizer, followed by a new centrifugation at 12,500 rpm for 15 min. The supernatant was considered the soluble fraction containing TR. Protein concentrations (mg/mL) were determined in a microplate spectrophotometer, with readings at 260 and 280 nm. The samples were stored at −70 °C until use.

The effect of SB-83 on trypanothione reductase (TR) activity was evaluated in 96-well plates. The soluble fraction (1 mg/mL) was incubated with SB-83 at concentrations equivalent to 1×, 2×, and 4× the IC_50_ of the compound for 6 min. Subsequently, an assay mixture containing HEPES (40 mM, pH 7.5), EDTA (1 mM), NADPH (100 μM), and trypanothione disulfide [T(S)_2_] (100 μM) was added. NADPH levels were then monitored by measuring absorbance at 340 nm using a spectrophotometer. Increased absorbance values indicate reduced NADPH consumption and lower TR activity. Controls consisted of reaction mixtures containing T(S)_2_ without SB-83 and supplemented medium as blank [24].

### 2.9. Molecular Anchoring

#### Computational Details

The ligand SB-83 was selected for the present study due to its potential inhibitory activity against molecular targets of *Trypanosoma cruzi*, as previously suggested in pharmacological investigations. Thus, the *T. cruzi* receptor corresponding to the trypanothione reductase (TR) protein, which is one of the main therapeutic targets for Chagas disease treatment, was employed to assess its interaction. The molecular structure of SB-83 was drawn directly using the Desmond module interface within Maestro^®^ v. 2023.1 (Schrödinger suite, academic license provided by D. E. Shaw Research, https://www.deshawresearch.com/resources.html (accessed on 9 May 2025) and subsequently energy-minimized using the standard OPLS_2005 [25], force field in order to generate an energetically stable three-dimensional conformation. The ligand was then exported in *.MOL2 format for subsequent molecular docking studies. The three-dimensional structure of TR (PDB ID: 1BZL) was retrieved from the RCSB Protein Data Bank (https://www.rcsb.org/) (accessed on 9 May 2025). All co-crystallized ligands present in the active sites were removed to expose the binding cavities.

TR possesses three relevant inhibitor-binding sites: (i) the Z site, a hydrophobic region located near the catalytic site and the NADPH cofactor binding site; (ii) the mepacrine site, a hydrophobic pocket located at the entrance of the trypanothione disulfide binding site; and (iii) the catalytic site, where trypanothione disulfide reduction occurs. Molecular docking was performed using the GOLD® software v. 5.8.1 (Cambridge, Cambs, UK), in which the search box was defined for each docking simulation based on the specific coordinates of each binding site: catalytic site (X = 24.663, Y = −4.301, Z = 9.125), mepacrine site (X = 17.733, Y = −1.197, Z = 13.499), and Z site (X = 41.825, Y = 4.351, Z = −28.343). The top-ranked binding pose in each site was selected based on GoldScore ranking and visual inspection of the molecular interactions. This was then used as the initial structure for molecular dynamics (MD) simulations. These simulations were conducted using the Desmond module integrated into Maestro^®^ v. 2023.1, employing the OPLS_2005 force field. The system was prepared with the Protein Preparation Wizard, with protonation states of ionizable residues adjusted to physiological pH 7.4 using the PROPKA module. Each system was solvated in an orthorhombic box of explicit water molecules using the TIP3P model, maintaining a minimum distance of 10 Å between the protein and the box edges. Following energy minimization to resolve steric clashes, the systems were equilibrated under NPT conditions (constant number of particles, pressure, and temperature) at 300 K and 1 bar. Next, 100 ns simulations were performed for each complex with a recording interval of 100 ps, yielding approximately 1000 frames per simulation. Post-simulation analyses included evaluating the conformational stability of the complexes through root-mean square deviation (RMSD) of the protein-ligand complexes, as well as monitoring the interactions between TR and SB-83 via interaction diagrams and occupancy fraction plots, enabling us to identify residues with the highest interaction persistence [26]. Additionally, root mean square fluctuations (RMSF) of the protein residues were calculated to assess local flexibility of the polypeptide chain in response to SB-83 binding.

Binding free energies (Δ*G_bind_*) were computed using the MM/GBSA method [27] via the Prime MM/GBSA module in Maestro^®^, applying the OPLS_2005 force field. Thus, 10 representative frames extracted from the most stable simulation phase (corresponding to a 10 ns interval) were used for each system to estimate binding affinities [28]. The binding free energy was estimated using the following equation:

Δ*G_lig_* = Δ*E_MM_* + Δ*G_solv_* − Δ*G_SA_*

In which Δ*E_MM_* = *E_complex_* − (*E_protein_* + *E_ligand_*); Δ*G_Solv_* = Δ*G_SolvComplex_* − (Δ*G_SolvProtein_* + Δ*G_SolvLig_*); then, Δ*G_SA_* = Δ*G_SAComplex_* − (Δ*G_SAProtein_* + Δ*G_SALigand_*). Moreover, *Solv* means solvation energy; *SA* represents the surface are energy; and *E_MM_* means minimized energy values for the complex (*E_complex_*), with ligands (*E_lig_*) and (*E_protein_*) [29,30].

### 2.10. Ultrastructural Evaluation

*T. cruzi* epimastigotes (10^7^ cells/mL) were incubated for 24 h at 26 °C in two groups: control (culture medium) and samples treated with SB-83 (1 × IC_50_). After incubation, the samples were washed three times with PBS (centrifugation at 3500 rpm, 10 min) and fixed with Karnovsky (2% glutaraldehyde, 2% paraformaldehyde in 0.1 M cacodylate buffer, pH 7.2) for 3 h. Post-fixation was performed in 1% osmium tetroxide, 0.8% potassium ferricyanide and 5 mM CaCl_2_. The samples were dehydrated with acetone (30% to 100%).

The TEM samples were embedded in Spurr resin, cut in an ultramicrotome (Leica Microsystems, Wetzlar, Germany), contrasted with uranyl acetate and lead citrate, and analyzed in a JEOL JEM-1011 microscope (JEOL, Peabody, MA, USA). In turn, the SEM samples were subjected to critical point drying with CO_2_ (CPD 030, BALZERS, Agawam, MA, USA) and metallized with gold (Leica EM SCD 550, LEICA, Wetzlar, Germany). Morphological analyses were performed in a JEOL JSM-7001F microscope (Jeol, Tokyo, Japan) [31,32].

### 2.11. Cell Death Profile Assessment

First, 1 × 10^7^ trypomastigotes were cultured in 24-well plates and treated for 24 h with SB-83 concentrations equivalent to 1×, 2× and 4× the IC_50_. After treatment, the parasites were washed three times with PBS and resuspended in binding buffer containing HEPES (10 mM), NaCl (140 mM) and CaCl_2_ (2.5 mM), adjusted to pH 7.4. Labeling was performed using a commercial Annexin V-FITC/IP apoptosis detection kit, according to the manufacturer’s instructions. The labeled samples were incubated for 15 min and then diluted in binding buffer containing Annexin V. Analysis was performed on a BD FACSCanto^®^ II flow cytometer (BD Biosciences, San Jose, CA, USA), monitoring fluorescence in a total of 30,000 events. Data were processed and analyzed using FlowJo 10.0.7 software (TreeStar Inc., Ashland, OR, USA) [18].

### 2.12. Statistical Analysis

The experiments were performed in triplicate and five independent experiments. The mean concentrations (CC_50_ and IC_50_) were determined by nonlinear regression. The differences between the experimental groups were evaluated by analysis of variance (ANOVA), followed by Tukey’s multiple comparisons test, considering statistical significance for *p* < 0.05.

## 3. Results

### 3.1. Evaluation of Anti-Trypanosoma Activity Against Epimastigote and Trypomastigote Forms of Trypanosoma cruzi

**Figure 2:**
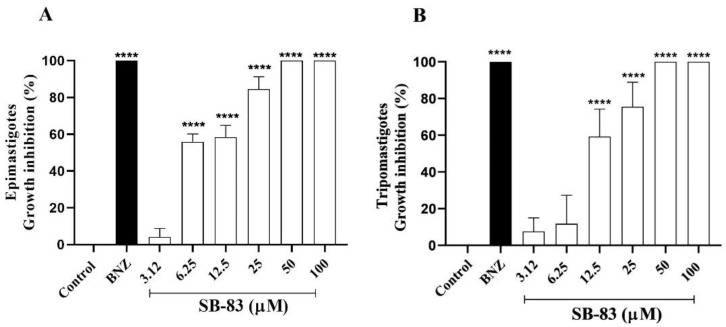
Inhibitory action of SB-83 on epimastigote (**A**) and trypomastigote (**B**) forms of *Trypanosoma cruzi*. The Figure represents the mean ± standard error of the mean of five independent experiments considering the control (complete LIT medium for epimastigotes and DMEM for trypomastigotes) as 0% inhibition, and benznidazole (BNZ) at a concentration of 200 µM was used as a positive control. One-way ANOVA was performed followed by Tukey’s post-test for comparison between groups, with (****) *p* < 0.0001 compared to the control.

The evaluation of the thiophene derivative SB-83 against epimastigote forms of *T. cruzi* demonstrated significant inhibitory effects, with reductions of 55.92% at 6.25 µM, 58.49% at 12.5 µM and 84.56% at 25 µM (Figure 2A). Complete inhibition (100%) of the growth of epimastigote forms was observed at concentrations of 50 µM and 100 µM (Figure 2A). The calculated IC_50_ value was 6.23 ± 0.84 µM (Table 1). For comparison, the reference drug benznidazole (200 µM) was also evaluated, demonstrating 100% inhibition of epimastigote forms with IC_50_ of 111.81 ± 2.1 µM (Table 1).

SB-83 demonstrated a significant inhibitory capacity in the evaluation of the anti-Trypanosoma activity of the growth of trypomastigote forms, reducing the viability of the forms by 59.29% at 12.5 µM and 75.54% at 25 µM (Figure 2B). Complete inhibition (100%) of the growth of trypomastigote forms was observed at the two highest concentrations (50 µM and 100 µM), with IC_50_ of 7.31 ± 0.52 µM (Table 1). Benznidazole showed total inhibition (100%) of trypomastigote forms with IC_50_ of 21.11 ± 1.1 µM (Table 1).

### 3.2. Evaluation of Cytotoxicity Against LLC-MK2 CCL-7 and Vero CCL-81 Cell Lines

**Figure 3:**
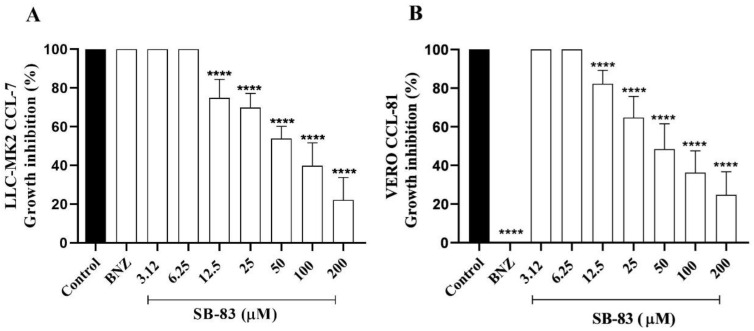
Effect of SB-83 on the viability of LLC-MK2 CCL-7 (**A**) and Vero CCL-81 (**B**) cell lines. Data represent the mean ± standard error of the mean of at least five independent experiments performed in triplicate, considering the negative control (complete DMEM without treatment) as 100% viability, and Benznidazole (BNZ) at a concentration of 200 µM was used as the positive control. Comparison between groups was performed by One-way ANOVA followed by Tukey’s post-test, with (****) *p* < 0.0001 compared to the control.

The SB-83 compound showed cytotoxicity against LLC-MK2 and Vero cells at concentrations ranging from 12.5 to 200 µM. Cell viability for LLC-MK2 cells was reduced by 21.91%, 30.05%, 46.15%, 57.31%, and 77.84% at concentrations of 12.5 µM, 25 µM, 50 µM, 100 µM, and 200 µM, respectively (Figure 3A). The observed reductions in Vero cells were 18.06%, 35.48%, 49.28%, 62.73%, and 75.23% at the same concentrations (Figure 3B). The CC_50_ values were 77.80 ± 2.05 µM for LLC-MK2 and 24.21 ± 1.2 µM for Vero (Table 1).

Benznidazole presented a CC_50_ value greater than 200 µM for LLC-MK2 and for Vero cells, while benznidazole presented a CC_50_ value of 147.37 ± 2.74 µM (Table 1). The selectivity index (SI) of SB-83 was higher than that of benznidazole, with values of 12.49 and 3.89 (epimastigotes) and 10.64 and 3.31 (trypomastigotes) for LLC-MK2 and Vero, respectively (Table 1).

### 3.3. Anti-Trypanosoma cruzi Activity Assay Against Intramacrophagic Amastigote Forms

**Figure 4:**
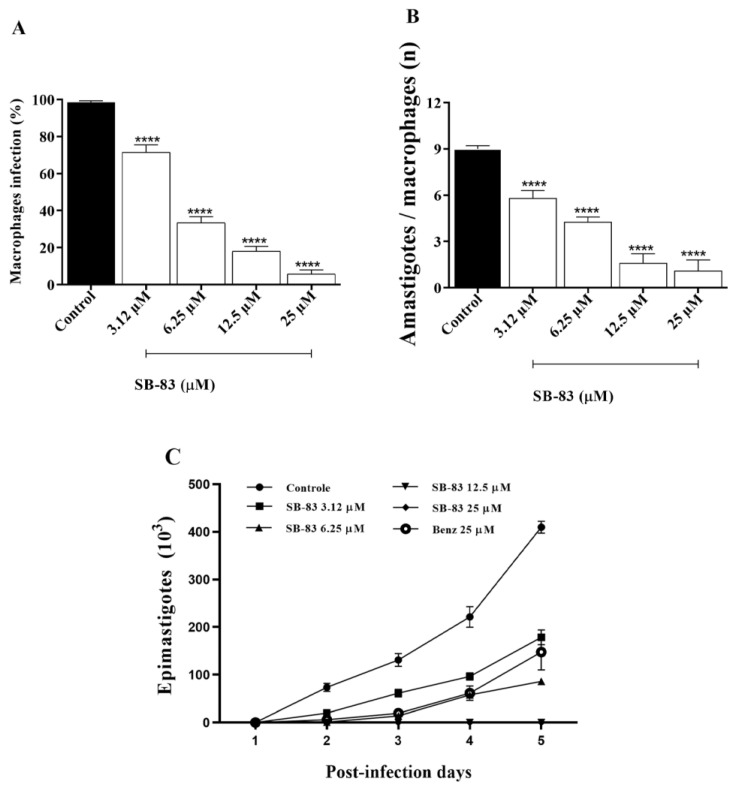
Anti-*Trypanosoma cruzi* activity of SB-83 against intramacrophagic amastigotes. (**A**) Percentage of infected macrophages. (**B**) Number of amastigotes per macrophage. (**C**) Recovery of epimastigotes after differentiation of trypomastigotes released from SB-83–treated infected macrophages and subsequent culture in LIT medium. Data represent the mean ± standard error of the mean of five independent experiments performed in triplicate, considering the control (DMSO 0.5% in complete DMEM) as 0% inhibition and benznidazole (BNZ) at 25 µM as a positive control. Comparisons between groups were performed using one-way ANOVA followed by Tukey’s post-test, with (****) *p* < 0.0001 compared to the control.

SB-83 demonstrated a significant reduction in the percentage of infected macrophages at all concentrations tested (3.12 μM, 6.25 μM, 12.5 μM, and 25 μM), with reductions of 27.09%, 65.2%, 80.53%, and 92.82%, respectively, compared to the negative control (Figure 4A). Furthermore, the compound significantly reduced the number of amastigotes per macrophage, with decreases of 35.44%, 52.48%, 82.22%, and 87.78% at the same concentrations (Figure 4B).

In addition, the activity of SB-83 against intracellular amastigote forms was indirectly evaluated by quantifying the recovery of epimastigotes after differentiation of trypomastigotes released from treated infected macrophages. Under these conditions, SB-83 completely suppressed parasite recovery at concentrations of 12.5 μM and 25 μM, with no epimastigote growth detected after 5 days of culture (Figure 4C). At lower concentrations (3.12 μM and 6.25 μM), reductions of 56.38% and 79.01% were observed on day 5, respectively. In comparison, benznidazole (25 μM) reduced parasite recovery by 63.92%, indicating higher efficacy of SB-83 against intracellular forms.

### 3.4. Cytokine Expression Assessment

**Figure 5:**
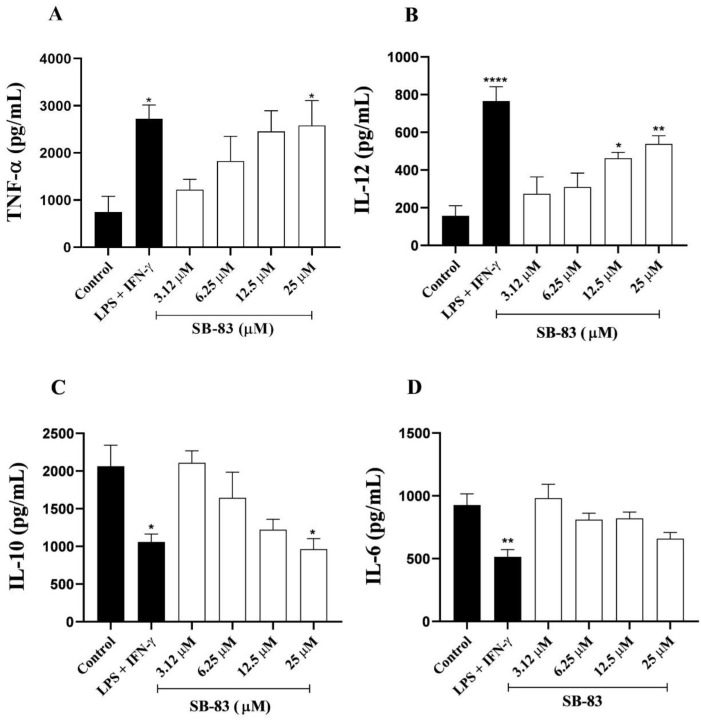
Cytokine levels produced by macrophages infected with *Trypanosoma cruzi* and treated with SB-83. (**A**) TNF-α; (**B**) IL-12; (**C**) IL-10; (**D**) IL-6. Data represent the mean ± standard error of the mean. Comparison between groups was performed by One-way ANOVA followed by Tukey’s post-test, where (*) *p* < 0.05, (**) *p* < 0.01 and (****) *p* < 0.0001 compared to the control.

Considering the anti-amastigote activity of SB-83, its immunomodulatory impact in the supernatant of the infection assays was also evaluated. Thus, cytokine quantification in the supernatants revealed that SB-83 modulated the immune response in macrophages infected with *T. cruzi*. The treatment increased TNF-α levels by 245.28% at 25 μM (Figure 5A) and IL-12 levels by 194.25% and 242.28% at concentrations of 12.5 μM and 25 μM, respectively (Figure 5B). There was suppression of IL-10, with a reduction of 53.32% at 25 μM (Figure 5C), while IL-6 levels remained unchanged (Figure 5D).

### 3.5. Measurement of Nitric Oxide (NO) and Reactive Oxygen Species (ROS) Production

**Figure 6:**
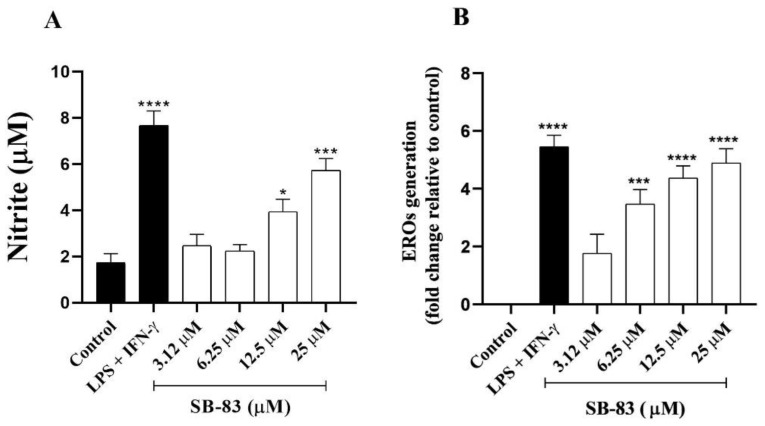
Nitrite (**A**) and reactive oxygen species (ROS) (**B**) production in macrophages infected with *Trypanosoma cruzi* and treated with SB-83. NO production was assessed by the Griess method and ROS production by 2′,7′-dichlorofluorescein diacetate (H2DCFDA). Data represent the mean ± standard error of the mean of at least five independent experiments performed in triplicate. Comparison between groups was performed by One-way ANOVA followed by Tukey’s post-test, where * *p* < 0.05; *** *p* < 0.001 and **** *p* < 0.0001 compared to the control.

Indirect evaluation of NO production based on quantification of nitrites in the supernatants of the infection assays revealed a significant increase in NO levels (an inflammatory mediator) after treatment with SB-83. The observed increase was 126.77% at a concentration of 12.5 μM and 230.61% at 25 μM when compared to the control (Figure 6A).

In addition, the analysis of ROS production in infected macrophages indicated an increasing response over 72 h of exposure to SB-83. ROS levels increased progressively, reaching 3.47%, 4.36% and 4.86% at concentrations of 6.25 μM, 12.5 μM and 25 μM, respectively (Figure 6B).

### 3.6. Effect of SB-83 on Trypanothione Reductase (TR) Activity

**Figure 7:**
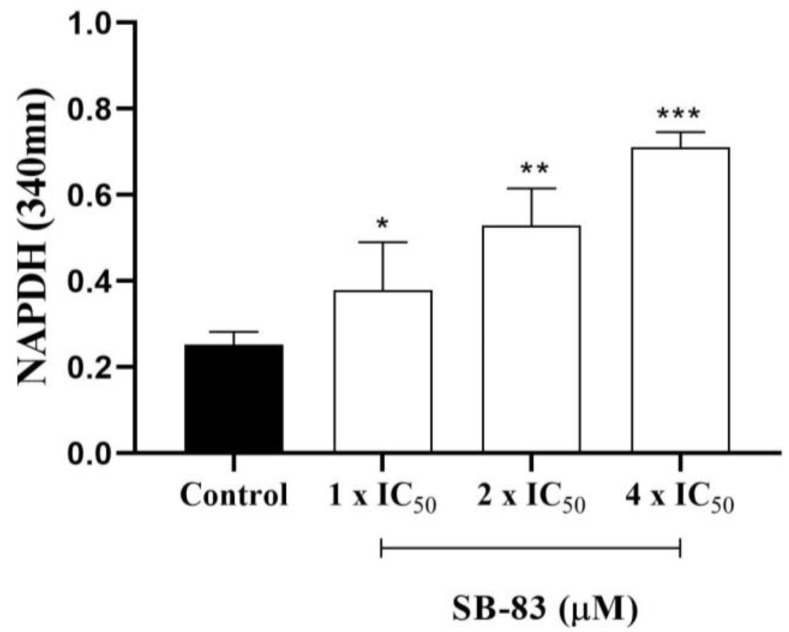
Effect of SB-83 on trypanothione reductase (TR) activity, evaluated by monitoring residual NADPH levels at 340 nm. Data represent the mean ± standard error of five independent experiments performed in triplicate. The control group contained the trypanothione disulfide substrate (T(S)_2_) without SB-83. Statistical significance was determined by one-way ANOVA followed by Tukey’s post-test (* *p* < 0.05; ** *p* < 0.01; *** *p* < 0.001 vs. control).

SB-83 promoted a concentration-dependent reduction in trypanothione reductase (TR) activity, as evidenced by increased residual NADPH levels compared to the control (Figure 7). Treatment with SB-83 at concentrations equivalent to 1×, 2×, and 4× IC_50_ resulted in progressively higher NADPH absorbance at 340 nm, indicating decreased NADPH consumption and reduced enzymatic activity. The highest concentration tested (4× IC_50_) produced the most pronounced reduction in TR activity.

### 3.7. Molecular Dynamics Simulations

The 100 ns molecular dynamics (MD) simulations provided relevant insights into the stability of the complexes formed between the SB-83 ligand and the different binding sites of the target protein. Overall, the ligand remained stable across all binding sites, whereas the protein’s dynamic behavior varied depending on the specific binding location.

**Figure 8:**
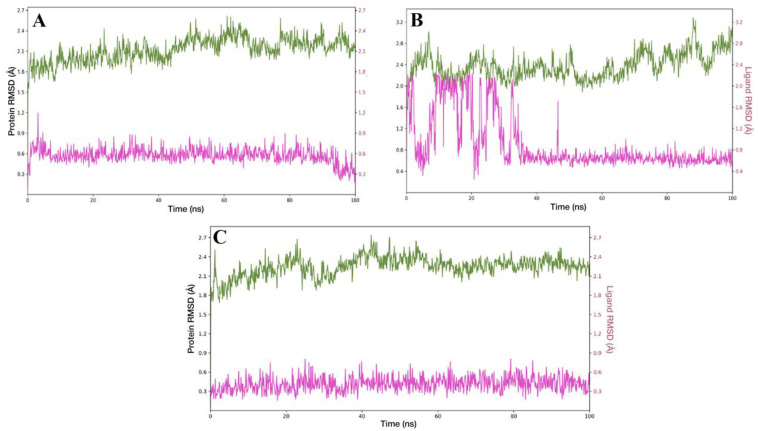
Root Mean Square Deviation (RMSD) of the trypanothione reductase–SB-83 complexes over 100 ns of molecular dynamics simulation: (**A**) catalytic site, (**B**) mepacrine site, and (**C**) Z site. The green and pink lines represent the RMSD plots for protein and ligand, respectively.

Both the protein backbone and the ligand exhibited consistent stability in the complex formed at the catalytic site throughout the simulation (Figure 8A). The RMSD of the SB-83–catalytic site ranged between 1.5 and 2.7 Å after a brief initial relaxation period, indicating a well-equilibrated system. SB-83 displayed an RMSD ranging from 0.3 to 0.7 Å with a linear trajectory over time, suggesting that the ligand remained firmly bound within the catalytic pocket. The protein showed more pronounced fluctuations for the SB-83–mepacrine complex compared to the catalytic site, with RMSD values ranging from 2.0 to 3.2 Å (Figure 8B). This instability was particularly evident during the first 20 ns, during which the ligand also exhibited high RMSD values (up to ~2.3 Å), suggesting a conformational adjustment phase. Despite these early fluctuations, the ligand remained anchored within the site. Then from 40 ns onward, the RMSD of SB-83 stabilized within the 0.4 to 0.8 Å range, indicating that the ligand eventually adopted and maintained a stable conformation within the mepacrine binding site.

The dynamic profile in the SB-83–Z complex closely resembled that of the catalytic site (Figure 8C). The protein backbone fluctuated moderately between 1.8 and 2.4 Å, while the ligand remained highly stable, with RMSD values gently oscillating between 0.3 and 0.6 Å, showing no signs of dissociation throughout the simulation. These results collectively suggest that SB-83 exhibits high conformational stability within all three evaluated binding sites, with particularly favorable profiles observed at the catalytic and Z sites, where both protein fluctuations and ligand displacement were minimal. These findings support the hypothesis that SB-83 is capable of effectively adapting to different microenvironments within the target, potentially enabling its activity at multiple binding sites.

**Figure 9:**
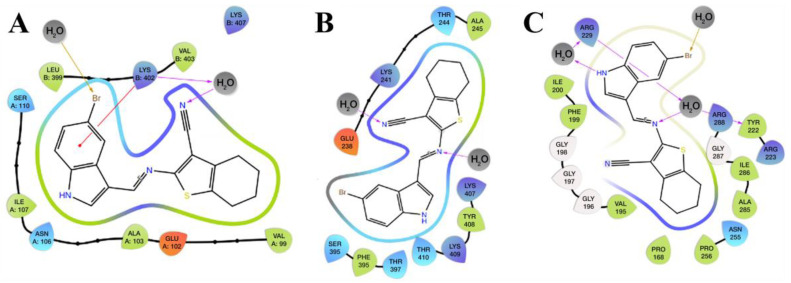
Two-dimensional interaction diagrams of SB-83-trypanothione reductase complexes: (**A**) catalytic site, (**B**) mepacrine binding site, and (**C**) Z-binding site. Colored arrows are used as visual guides to highlight ligand–residue interactions and do not represent different interaction types.

SB-83 established hydrophobic interactions at the catalytic site with VAL^99^, ALA^103^, ILE^107^, LEU^399^, and VAL^403^ residues, performing a predominantly nonpolar environment surrounding the ligand. Favorable electrostatic interactions were observed with LYS^402^ and LYS^407^, while polar contacts involved ASN^106^ and SER^110^. Additionally, a relevant interaction with the negatively charged residue GLU^102^ was identified. Moreover, two water molecules played critical structural roles: one interacted with the bromine atom of SB-83 via halogen bonding, while the other formed a hydrogen bond with the nitrogen atom of the ligand’s alkyne group and simultaneously interacted with LYS^402^, which also engaged in a cation–π interaction with the aromatic ring of SB-83 (Figure 9A). Although the docking grid was correctly centered over the TR catalytic site, SB-83 stabilized in a region adjacent to the key catalytic residues (Cys^53^, Cys^58^, His^461^, Glu^466^, and Glu^467^), which are directly involved in the enzyme’s reductive activity [33,34]. Therefore, SB-83 appears to act as an allosteric or entrance-site inhibitor, blocking access of trypanothione to the catalytic center rather than acting as a direct competitive inhibitor. This alternative inhibition mechanism is pharmacologically plausible, as steric hindrance of substrate access to the active site may reduce TR activity, constituting a strategy also suggested for other compounds in previous studies [35]. Hydrophobic interactions were predominant at the mepacrine–SB-83 site involving ALA^245^, PHE^395^, and TYR^408^ residues, along with polar contacts with THR^244^, SER^395^, THR^397^, and THR^410^. The ligand also formed electrostatic interactions with positively charged LYS^241^, LYS^407^, and LYS^409^ residues, as well as with the negatively charged GLU^238^. Moreover, two water molecules also contributed to ligand stabilization: one interacted with the nitrogen of the alkyne group of SB-83, while the other contacted the nitrogen of the ligand’s open chain (Figure 9B). This interaction pattern is consistent with those reported for other inhibitors targeting the TR entrance site, as described by Gomez-Escobedo [35], reinforcing the potential of SB-83 as a competitive inhibitor at this site.

SB-83 engaged in key hydrophobic interactions at the Z site with PRO^168^, VAL^195^, PHE^199^, ILE^200^, ALA^205^, TYR^222^, PRO^256^, and ILE^286^ residues. Significant polar interactions occurred with ASN^255^, and electrostatic interactions were observed with ARG^223^, ARG^229^, and ARG^288^; these residues have been reported to be critical for ligand recognition at the Z site [35]. Furthermore, the ligand interacted with four glycine residues (GLY^196^, GLY^197^, GLY^198^, and GLY^287^), which may contribute to local flexibility of the binding pocket, facilitating SB-83 accommodation. Finally, three water molecules participated in key interactions: one bridged the ligand’s NH group to ARG^229^; another linked the nitrogen of the ligand’s open chain to TYR^222^; and a third engaged in halogen bonding with the bromine atom of SB-83 (Figure 9C). This interaction network, including solvent-mediated contacts, is characteristic of high-affinity compounds targeting the Z site, as demonstrated in docking and molecular dynamics studies by [35]. Accordingly, the molecular interactions observed for SB-83 substantially overlap with the critical residues reported in the literature [33,34], validating its affinity for the major binding sites of trypanothione reductase. These findings collectively support the potential of SB-83 as a promising trypanocidal agent.

### 3.8. Binding Free Energy Calculated Using the MM/GBSA Method

The binding free energy data calculated using the MM/GBSA method (Table 2) provides a quantitative assessment of the affinity between the SB-83 ligand and the three TR binding sites and is based on representative frames extracted at regular intervals throughout the 100 ns MD simulations. These results enable us to identify specific interaction patterns and energetic contributions that support the stability of the complexes.

The complex formed at the Z-site exhibited the most favorable binding free energy with a Δ*G* value of −61.45 ± 8.62 kcal/mol, indicating strong and stable interactions within this hydrophobic region adjacent to the NADPH-binding site. These findings suggest that SB-83 efficiently accommodates within this site, likely due to extensive hydrophobic contacts. The complex also displayed a notably favorable binding energy at the catalytic site, with a Δ*G* value of −44.64 ± 4.39 kcal/mol, reinforcing the ligand’s ability to directly interact with the region responsible for trypanothione disulfide reduction. The stability of this complex is consistent with the functional relevance of this site for enzymatic activity. The mepacrine-binding site showed the least favorable binding energy among the three, with a Δ*G* value of −38.09 ± 1.77 kcal/mol. Nonetheless, this value still reflects a reasonably stable interaction, although it suggests an energetic preference of SB-83 for the Z-site and the catalytic site over the mepacrine site. Overall, the MM/GBSA results align with the RMSD and RMSF data obtained from the MD analyses, supporting the observed complex stability and indicating preferential binding of SB-83 to the Z-site of TR enzyme.

### 3.9. Evaluation of the Cell Death Profile of SB-83

**Figure 10:**
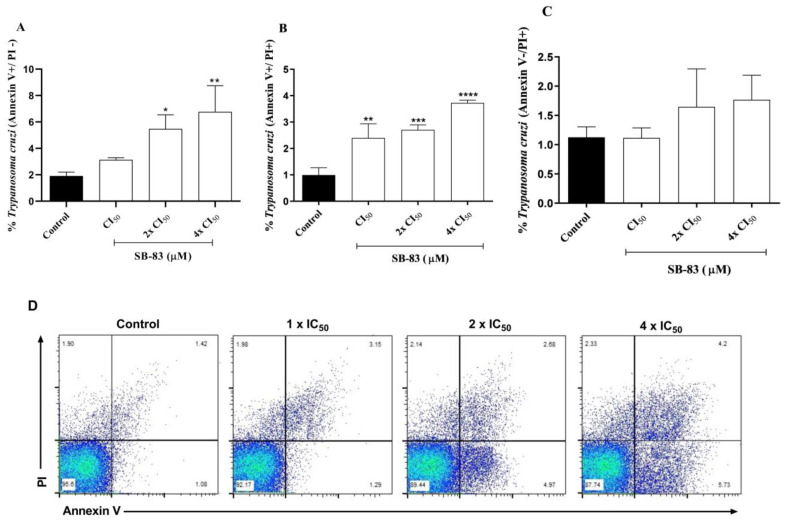
Evaluation of the death profile of *Trypanosoma cruzi* trypomastigote forms treated with SB-83 by flow cytometry. The parasites were labeled with Annexin V-FITC and Propidium Iodide (PI) and treated for 24 h with SB-83 concentrations of 1 × IC_50_, 2 × IC_50_ and 4 × IC_50_. (**A**) annexin V-FITC+/PI- staining patterns; (**B**) annexin V-FITC+/PI+ staining patterns; (**C**) annexin V-FITC-/PI+ staining patterns; and (**D**) representative dot plots showing staining of *T. cruzi* trypomastigotes. The data represent the mean ± standard error for five independent experiments performed in triplicate, considering the control group containing only the trypomastigote forms without drugs. (*) *p* < 0.05 vs. control; (**) *p* < 0.01 vs. control; (***) *p* < 0.001 vs. control; (****) *p* < 0.0001 vs. control.

The assay to evaluate the cell death profile of the SB-83 compound against trypomastigote forms was performed using the flow cytometry technique. The results showed a significant increase in Annexin V expression in the parasites treated with the concentrations of 2 × IC_50_ and 4 × IC_50_, with significance levels of 95% (*p* < 0.05) and 99% (*p* < 0.01), respectively, when compared to the control (Figure 10A). It was observed in Figure 10B (which represents the double Annexin V^+^-PI^+^ labeling) that the parasites treated with SB-83 exhibited an increase in all concentrations tested, with the highest expression observed in the concentration of 4 × IC_50_, presenting a significance of 99.99% (*p* < 0.0001). On the other hand, in Figure 10C, it was verified that the treated parasites did not present significant labeling for PI in any of the tested concentrations when compared to the control.

### 3.10. Structural Analysis of Epimastigote Forms

**Figure 11:**
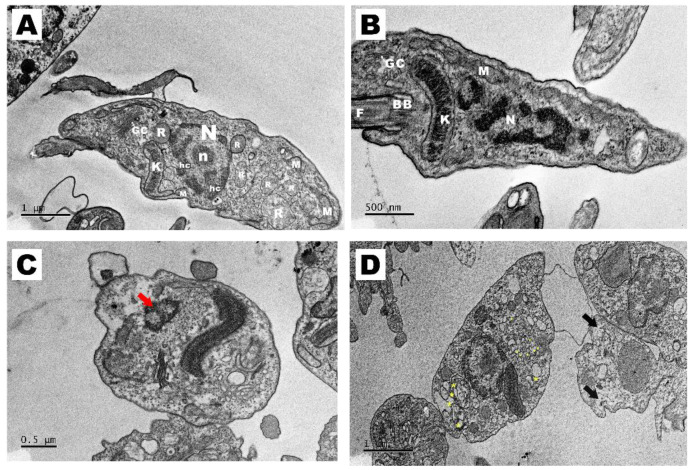
Effects of SB-83 on the morphology of *Trypanosoma cruzi* epimastigotes using Transmission Electron Microscopy (TEM). Epimastigotes were plated in complete LIT medium, treated with 15 μM SB-83 for 24 h and analyzed by TEM. (**A**,**B**) Negative control, showing typical elongated morphology of the parasite and normal organelles. (**C**,**D**) Treatment with 15 μM SB-83, showing rounding of the parasite, cellular disorganization and many electron-lucent vacuoles. M = mitochondria, N = nucleus, n = nucleolus, F = flagellum, K = kinetoplast, hc = heterochromatin; GC = Golgi complex; R = Reservosomes; BB = Basal body, * (yellow) = vacuoles. Red arrow = chromatin condensation; Black arrow = increase in reservosomes.

The structural alterations induced by SB-83 in *T. cruzi* epimastigote forms were analyzed by Transmission Electron Microscopy (TEM). The parasites presented preserved morphology (Figure 11A,B—untreated control), with an elongated body and intact structures, including cell membrane, nucleus, kinetoplast, Golgi complex and mitochondria.

In contrast, Figure 11C,D show significant structural alterations after treatment with SB-83. Figure 11C shows a reduction in nuclear size, chromatin condensation (red arrow) and rounding of the cell body accompanied by structural disorganization. Figure 11D reveals an increase in granules, proliferation of vacuoles and intracellular vesicles.

**Figure 12:**
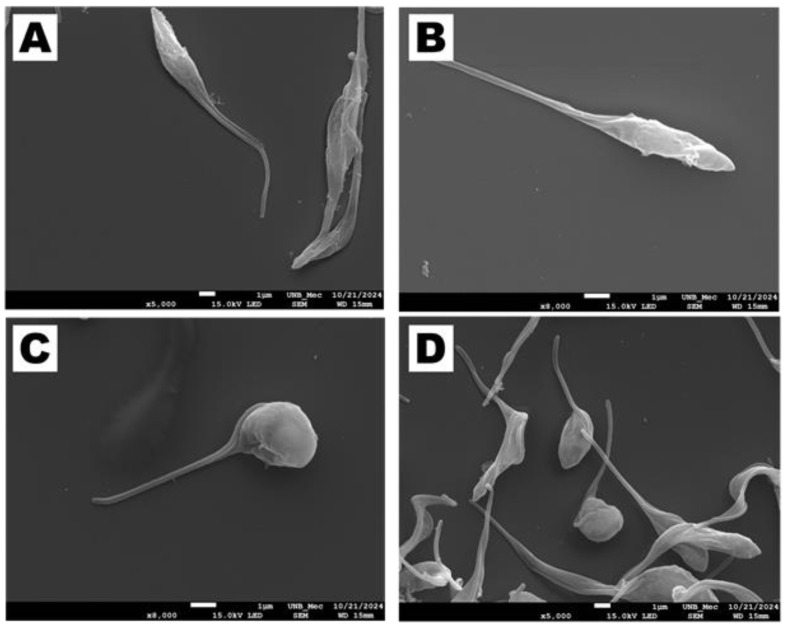
Effects of SB-83 on the morphology of *Trypanosoma cruzi* epimastigotes using Scanning Electron Microscopy (SEM). Epimastigotes were plated on complete LIT medium, treated with 15 μM SB-83 for 24 h, and analyzed by SEM. (**A**,**B**) Negative control showing typical elongated morphology of the parasite. (**C**,**D**) Treatment with 1 × IC_50_ of SB-83, showing rounding of the parasite.

On the other hand, Scanning Electron Microscopy (SEM) analysis revealed significant morphological changes in cells treated with SB-83. Untreated cells (Figure 12A,B) presented preserved morphology, with an elongated and thin body and intact flagellum. In contrast, parasites treated with SB-83 (Figure 12C,D) exhibited significant morphological changes, such as cell membrane deformation, as well as cell body rounding, shortening and twisting, resulting in evident loss of the typical structure.

## 4. Discussion

The anti-*Trypanosoma cruzi* activity of the SB-83 compound was evaluated against epimastigote, trypomastigote and intracellular amastigote forms of the parasite, demonstrating significant inhibition of parasite growth, even at low concentrations. The results obtained corroborate previous studies that highlighted thiophene derivatives as potential trypanocidal agents. For example, Gerpe et al. [36] reported an IC_50_ of 6.4 µM for a 5-nitrothiophene derivative in epimastigotes, which is similar to that observed in this study. Similarly, Silva-Júnior et al. [37] observed an IC_50_ of 10.3 µM for trypomastigotes with thiophene derivatives. Furthermore, SB-83 exhibited lower IC_50_/EC_50_ values than benznidazole across all parasite forms evaluated, with the highest potency observed against intracellular amastigotes, supporting its potential as a candidate for further investigation as an antiparasitic agent.

The cytotoxicity of SB-83 was evaluated in LLC-MK2 and Vero renal epithelial cells, presenting higher CC_50_ values than the IC_50_ values observed for *T. cruzi*. This profile highlights the compound’s ability to reach the parasite at concentrations tolerable by the host cells. Comparisons with previous studies reinforce the safety of thiophene derivatives in biological applications. In the study by Saha et al. [38], Thiophenic derivatives such as N1,N5-Dihexyl-2-(thiophene-2-sulfonamido)pentanediamide (VIg) showed a CC_50_ value of 27.12 µM in Vero cells, demonstrating similar toxicity to SB-83 [31]. In comparison, derivative 5A, described by Rodriguez et al. [39], presented a CC_50_ value of 17.69 µM in LLC-MK2 cells, indicating greater toxicity compared to SB-83 in this study.

The selectivity index (SI) calculated for SB-83 exceeded 10 when LLC-MK2 cells were used as the host cell model, indicating a favorable selectivity profile under these experimental conditions. Notably, higher SI values were observed for intracellular amastigote, highlighting preferential activity against the clinically relevant stage of *T. cruzi*. According to Peña et al. [40], SI values above 10 in in vitro assays may suggest preferential toxicity toward the parasite relative to host cells; however, this parameter is strongly dependent on the mammalian cell line employed. Indeed, when other cell lines were considered, lower SI values were observed for SB-83, underscoring the cell-dependent nature of its selectivity profile.

Our research group additionally evaluated the cytotoxicity of SB-83 in macrophages from the RAW 264.7 cell lines and murine peritoneal macrophages (Swiss), with CC_50_ values of 52.72 µM and 113.4 µM, respectively [8,9]. The selectivity index (SI) was calculated based on the CC_50_ value for RAW 264.7 previously described by Rodrigues et al. [9] and the antiparasitic activity data obtained in this study for SB-83, which presented values of 8.39 for epimastigote forms and 7.15 for trypomastigotes. These findings indicate that SB-83 exerts antiparasitic activity at significantly lower concentrations than its toxic dose for 50% of host cells, enabling evaluation of its efficacy against intracellular amastigote forms.

Given the selective efficacy of SB-83 against epimastigotes and trypomastigotes, its activity against intracellular amastigote forms was investigated since these are the forms responsible for chronic infection and irreversible tissue damage, especially in the myocardium and digestive tract [41]. Thus, the treatment significantly reduced the percentage of infected cells and the parasite load, demonstrating the ability of SB-83 to eliminate these replicative forms. These findings are corroborated by the results of the recovery assay, which revealed a significant reduction in the growth of epimastigote forms, evidencing the efficacy of SB-83 in eliminating intracellular amastigotes.

These findings are in line with previous studies describing the selectivity of thiophene derivatives. In the study by Rodrigues et al. [8], the authors evaluated SB-83 against intracellular amastigote forms of *Leishmania amazonensis*, obtaining an EC_50_ of 6.4 µM. However, SB-83 demonstrated greater efficacy against *T. cruzi* in the present study, presenting a lower EC_50_ value (5.12 ± 0.84 µM) compared to *T. cruzi* amastigotes. Similarly, Pacheco et al. [42] reported an EC_50_ of 5.42 ± 1.32 µM for the thiophene derivative 5-nitrothiophen-2-yl-N-t-butyl nitrone, constituting a value close to that obtained for SB-83, reinforcing its potential as a therapeutic candidate. Additionally, Silva-Júnior et al. [37] evaluated the activity of thiophene derivatives against amastigote forms of *T. cruzi* and reported an EC_50_ between 6.0 and 9.0 µM, highlighting the greater potential of SB-83 in eliminating these intracellular forms. These variations may be related to structural differences between the compounds evaluated, as well as to the specific susceptibility of each parasitic species.

The elimination of *T. cruzi* amastigotes is directly associated with macrophage activation and modulation of the immune response, involving pro-inflammatory mediator production such as TNF-α, IL-6 and IL-12, in addition to NO and ROS production, which are essential for controlling the infection [43]. However, excessive ROS accumulation can induce oxidative stress and mitochondrial dysfunction, contributing to develop chronic Chagas cardiomyopathy. Thus, homeostasis of the immune response regulated by anti-inflammatory cytokines such as IL-10 plays an essential role in limiting tissue damage [44].

Treatment with SB-83 resulted in a significant increase in TNF-α and IL-12 levels in this study, which are critical cytokines for the effector immune response against the parasite, without altering of IL-6 levels or reducing the IL-10 secretion. Furthermore, the compound stimulated NO and ROS production, suggesting a relevant immunomodulatory effect. However, the decrease in IL-10 levels may potentially compromise regulation of the inflammatory response, increasing the risk of tissue damage associated with exacerbated inflammatory processes. Previous studies have reported a similar effect of SB-83 in modulating the immune response against *L. amazonensis* and *L. infantum*, evidencing a conserved action pattern among parasites of the same family [8,28]. Despite the therapeutic potential of SB-83, its safety and impact on the immune response should be evaluated in additional studies in order to minimize possible adverse effects related to immune hyperactivation.

Next, the ability of SB-83 to inhibit TR, an essential enzyme exclusive to parasites of the Trypanosomatidae family, was investigated in order to elucidate its mechanisms of action. TR plays a crucial role in the parasite’s redox cycle, being responsible for protecting it against oxidative stress imposed by the host’s immune system [45]. This enzyme replaces the glutathione-glutathione reductase system present in human cells, providing a unique and indispensable metabolic pathway for the survival of trypanosomatids, such as *T. cruzi* and *Leishmania* spp. Thus, TR has been consolidated as one of the main targets for developing new therapeutic agents against neglected diseases, such as Chagas disease and leishmaniasis [34].

Therefore, in silico and in vitro analyses were performed to evaluate the effect of SB-83 against TR. In vitro analyses confirmed TR inhibition, evidenced by the increase in NADPH concentration, corroborating the potential of SB-83 as an effective enzyme inhibitor. Previous studies, such as those by Patterson et al. [46]; Zani and Fairlamb [47], also reported TR inhibition by thiophene derivatives (BTCP and TNQ2), consolidating the potential of this class of compounds.

In addition, the cell death mechanisms of SB-83 in *T. cruzi* were evaluated. A predominant death by apoptosis profile was observed, with greater significance in the late phase, supported by structural analyses of SEM and TEM which showed internal disorganization and external changes indicative of apoptosis, such as chromatin condensation, nuclear decrease and cell body shortening. However, there are no records of studies investigating the mechanism of death induced in *T. cruzi* by thiophenes and their derivatives in the literature. Nevertheless, related studies, such as Rodrigues et al. [8], who analyzed SB-83 in *Leishmania amazonensis*, and Carvalho et al. [11], Hess et al. [48], and Swain et al. [49], who investigated thiophene derivatives with anticancer action, reinforce that apoptosis may be a common mechanism of action for this class of compounds in different target cells.

## 5. Conclusions

In conclusion, the results of this study demonstrate the therapeutic potential of SB-83 as a selective and effective agent against the Colombian strain (DTU TcI) of *T. cruzi*. In addition, immune response modulation, TR inhibition and apoptosis induction suggest specific mechanisms of action, which may guide the development of new drugs based on thiophene derivatives. Therefore, the fact that DTU TcI is widely implicated in the cardiac pathogenesis of the infection further emphasizes the importance of these findings, suggesting that SB-83 may represent a viable candidate for future therapeutic investigations considering its effect on a strain with high clinical impact.

## Figures and Tables

**Table 1:** Anti-*Trypanosoma cruzi* action, cytotoxic activity against LLC-MK2 CCL-7, Vero CCL-8 and RAW 264.7 cell lines, and selectivity index (SI) values expressed for SB-83 and Benznidazole.

Compounds	MK2	Vero	RAW	Epimastigote				Trypomastigote				Intracellular Amastigotes			
	CC_50_ (µM)	CC_50_ (µM)	CC_50_ (µM)	IC_50_ (µM)	SI * MK2	SI * Vero	SI * RAW	EC_50_ (µM)	SI * MK2	SI * Vero	SI * RAW	EC_50_ (µM)	SI * MK2	SI * Vero	SI * RAW
**SB-83**	77.80 ± 2.05	24.21 ±1.2	52.27 ^a^	6.23 ± 0.84	12.49	3.89	8.39	7.31 ± 0.52	10.64	3.31	7.15	5.12 ± 0.84	15.19 ± 0.93	4.72	10.20
**Benznidazol**	>200	147.37 ± 2.74	79.23 ± 0.23	111.81 ± 2.11	>1.79	1.32	0.71	21.11 ± 1.1	>9.47	6.98	3.75	24.0 ± 2.11	>8.33	6.14	3.30

* SI (selectivity index) = CC_50_/IC_50_ or EC_50_. ^a^ Value calculated by Rodrigues et al. [9].

**Table 2:** Gibbs free energy of binding (ΔG) of SB-83 in complex with different binding sites of *T. cruzi* trypanothione reductase (TR), calculated using the MM/GBSA method.

Complex	Δ*G* ± SD (kcal/mol) ^a^	Δ*G* Range (kcal/mol) ^b^
SB-83-Z binding site	−61.45 ± 8.62	−79.79 to −53.76
SB-83-Catalytic site	−44.64 ± 4.39	−50.24 to −34.72
SB-83-Mepacrina site	−38.09 ± 1.77	−41.09 to −34.09

**^a^** Mean Gibbs free energy value ± standard deviation; ^b^ Range of maximum and minimum Gibbs free energy values.

## Data Availability

Dataset available on request from the authors.

## Supplementary Materials

The following supporting information can be downloaded at https://www.mdpi.com/article/10.3390/pathogens15010064/s1. Figure S1 HPLC chromatogram showing the purity of SB-83.
