# Supplementary material for: 2-Aminothiophene Derivative SB-83 Inhibits Trypanothione Reductase and Modulates Cytokine Production in Trypanosoma cruzi-Infected Cells
Source: Pathogens. 2026 Jan 8;15(1):64. doi: 10.3390/pathogens15010064 (PMC12845054; doi:10.3390/pathogens15010064)
Supplement: Supplementary file 1 [file pathogens-15-00064-s001.zip › pathogens-4041767-supplementary.pdf]

Supplementary data for

2-Aminothiophene Derivative SB-83 Inhibits Trypanothione Reductase and Modulates Cytokine Production in Trypanosoma cruzi-infected cells

Airton Lucas Sousa dos Santos [a], Vanessa Maria Rodrigues de Souza [a], Julyanne Maria Saraiva de Sousa [a], Raiza Raianne Luz Rodrigues [a], Mércya Lopes Braga [a], Maria Gabrielly Gonçalves Da Silva Sousa [a], Douglas Soares de Oliveira [a], Mirely Vitória Farias da Silva [b], Edeildo Ferreira da Silva-Junior [b], Thaís Amanda de Lima Nunes [c], Marcos Vinícius da Silva [c], Ingrid Gracielle Martins da Silva [d], Karine Brenda Barros-Cordeiro [d], Sônia Nair Báo [d], Francisco Jaime Bezerra Mendonça Junior [e] and Klinger Antonio da Franca Rodrigues [a,f*]

[a] Infectious Disease Laboratory, Campus Ministro Reis Velloso, Federal University of Piauí, 64202-020, Parnaíba, PI, Brasil

[b] Research Group on Biological and Molecular Chemistry, Institute of Chemistry and Biotechnology, Federal University of Alagoas, AC Simões campus, 57072-970, Maceió, Alagoas, Brazil.

[c] Laboratory of Immunology and Parasitology, Institute of Biological and Natural Sciences, Federal University of Triângulo Mineiro, Uberaba 38025-180, MG, Brazil

[d] Microscopy and Microanalysis Laboratory, Department of Cell Biology, Institute of Biological Sciences, University of Brasília, 70.910-900, Brasília, DF, Brazil

[e] Laboratory of Synthesis and Drug Delivery, Department of Biological Sciences, State University of Paraíba, 58071-160, João Pessoa, PB, Brazil

f Center for Basic and Applied Immunology, Dom Delgado University City, Federal University of Maranhão, 65065-545, São Luiz, MA, Brazil

* Correspondence: **Klinger Antonio da Franca Rodrigues**, Center for Basic and Applied Immunology, Dom Delgado University City, Federal University of Maranhão, 65065-545, São Luiz, MA, Brazil; Tel: +55 83 981836622 Email: klinger.antonio@ufma.br

Analysis Report

<Sample Information>

Sample Name	: SINTESE 1_A_conc.		
Sample ID	: SINTESE 1_A_conc.		
Data Filename	: SINTESE 1_A_conc..lcd		
Method Filename	: Vitoria.lcm		
Batch Filename	:		
Vial #	: 1-1	Sample Type	: Unknown
Injection Volume	: 10 uL		
Date Acquired	: 09/09/2025 11:20:32	Acquired by	: System Administrator
Date Processed	: 09/09/2025 11:30:41	Processed by	: System Administrator

<Chromatogram>

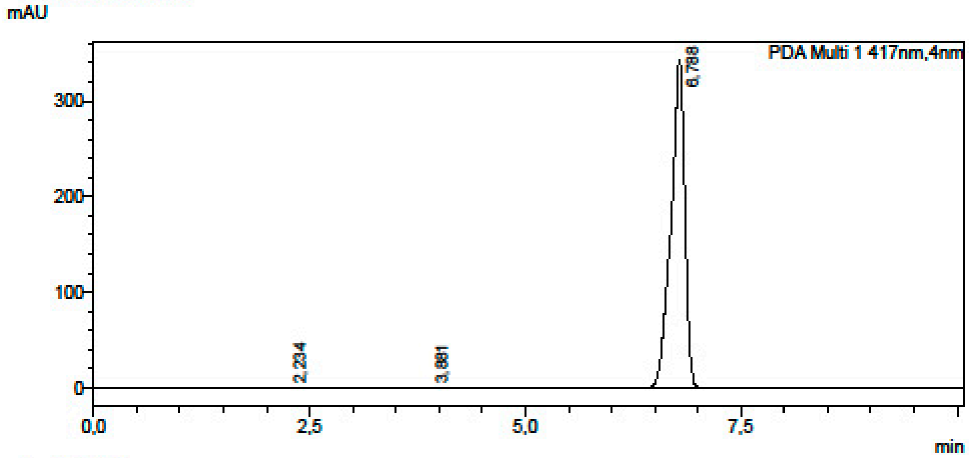

<Peak Table>

PDA Ch1 417nm

Peak#	Ret. Time	Area	Height	Conc.	Unit	Mark	Name
1	2,234	2586	654	0,068			
2	3,881	1203	163	0,031			
3	6,788	3824996	341973	99,901			
Total		3828784	342790				

Figure. S1 HPLC chromatogram showing the purity of SB-83.
